# Noninvasive monitoring of blood flow using a single magnetic microsphere

**DOI:** 10.1038/s41598-019-41416-6

**Published:** 2019-03-21

**Authors:** Maik Liebl, Bernhard Gleich, Dietmar Eberbeck, Patricia Radon, Jürgen Rahmer, Lutz Trahms, Frank Wiekhorst

**Affiliations:** 1 Physikalisch-Technische Bundesanstalt, Abbestraße 2-12, D-10587 Berlin, Germany; 2 Philips GmbH Innovative Technologies, Research Laboratories, Röntgenstraße 24-26, D-22315 Hamburg, Germany

## Abstract

Noninvasive medical imaging of blood flow relies on mapping the transit of a contrast medium bolus injected intravenously. This has the draw-back that the front of the bolus widens until the tissue of interest is reached and quantitative flow parameters are not easy to obtain. Here, we introduce high resolution (millimeter/millisecond) 3D magnetic tracking of a single microsphere locally probing the flow while passing through a vessel. With this, we successfully localize and evaluate diameter constrictions in an arteria phantom after a single passage of a microsphere. We further demonstrate the potential for clinical application by tracking a microsphere smaller than a red blood cell.

## Introduction

Cardiovascular disease is the number one cause of mortality with more than 17 million deaths per year worldwide^1^. One of the main causes of cardiovascular diseases are hemodynamic perturbations provoking the development of arterial stenosis. For early classification of such malfunctions, accurate and noninvasive measurement of vascular blood flow is required. A number of imaging modalities for diagnosis of hemodynamic disorders are available, such as coronary and peripheral angiography^2,3^, computed tomography (CT)^4,5^, magnetic resonance imaging (MRI)^6,7^, and, possibly in the future, magnetic particle imaging (MPI)^8^. All these tools have specific benefits, but unfortunately are also suffering from limitations that hamper their application as a screening tool in the clinical routine. These are limited temporal resolution^7^, exposure to ionizing radiation^9^, or the bolus administration of contrast agents possibly causing unwanted side effects^10^ and giving rise to uncertainties in flow quantification^11,12^. In noninvasive contrast enhanced MRI and CT, a bolus of contrast agents is injected intravenously and disperses in blood until the tissue of interest is reached. Hence, the front of the bolus widens considerably^13^ and differs from an ideal “sharp” bolus geometry desired for blood flow quantification^6^. In a semi-quantitative analysis, the dispersion of contrast agents is ignored, and the derived parameters obtained by the bolus transit do not necessarily have physiological correlates^6^. Other analysis techniques require mathematical models on the pharmacokinetics^14^ to derive quantitative parameters on blood flow. Further, the temporal resolution obtained by present MRI (20–50 ms)^15^ or CT (83–135 ms)^15^ scanners is limited by time-consuming spatial encoding schemes. The gold standard for blood flow quantification and stenosis detection are coronary and peripheral angiography^2,3^ with excellent temporal resolution of 1–10 ms^15^. Here, the contrast agents are injected close to the tissue of interest by a catheter. This is, however, an invasive procedure yielding nonnegligible risks for the patient^2,3^. Here, we present a novel approach named “Magnetic Microsphere Tracking” (MMT) that allows to circumvent these obstacles. By detecting the translational and rotational velocity of a single microsphere we can noninvasively quantify the local flow profile. With diameters down to less than 6 µm, the microspheres are small enough to pass through the cardio-vascular system without the risk of harmful side effects^16^. Magnetic tracking has so far been applied for magnetic entities of rather large dimensions (mm to cm in diameter)^17,18^. Extending this approach to micron-sized objects presented here opens new perspectives in diagnosis of cardiovascular disease.

**Figure 1:**
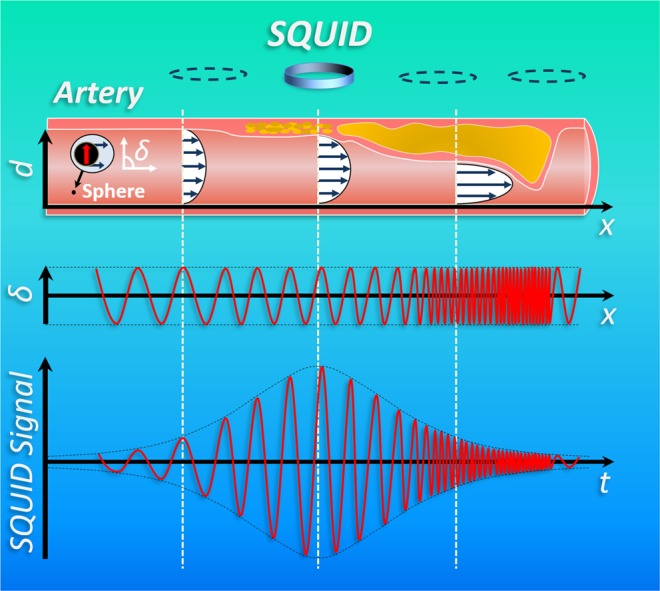
Magnetic microsphere with permanent dipole moment passing through an arteriosclerotic vessel (different degrees of stenosis). Top: The MM with permanent dipole moment passes a vessel with different grades of stenosis (arteriosclerotic plaques). Hence, the vessel diameter *d* is increasingly reduced along the *x*-coordinate and the velocity vectors of the quadratic flow profile increase. Middle: Sinusoidally changing sphere orientation, described by the azimuthal angle *δ* between the MM moment and the *x*-coordinate. Due to the diameter reduction and increasing shear forces the frequency *f*_rot_ of the sinusoidal signal will increase along *x*. Bottom: Resulting SQUID signal detecting the magnetic field of the MM over time *t*. The amplitude of the SQUID signal depends on the relative position between MM and SQUID showing maximum amplitude at shortest distance.

The principle of MMT is depicted in Fig. 1, a magnetic microsphere (MM) with permanent dipole moment moving inside a vessel in a laminar flow with quadratic profile is shown. The drag force generated by the flow translates and the shear force rotates the MM due to local velocity variations across the cross-section of the sphere.

These movements of the MM lead to variations of its magnetic dipole field that are remotely detected by an array of superconducting quantum interference devices (SQUIDs). In a vessel of constant diameter *d*, a steady motion would be the consequence with the orientation of the MM sinusoidally changing with time, so that the azimuthal angle *δ* is periodically oscillating along the *x-*coordinate at a constant frequency. Consequently, the SQUID detector will measure a sinusoidal signal over time at a unique frequency determined by the shear forces on the sphere. The amplitude of the SQUID signal depends on the MM position relative to the sensor, providing information that can be used for spatial decoding (see below). If a vessel suffers from stenosis (arteriosclerotic plaques), its cross section will be reduced increasing the velocity vectors of the flow profile. This leads to an acceleration of the translational and rotational motion of a MM passing the stenosis. Hence, the frequency of the sinusoidal SQUID signal increases and indicates the presence of the constriction. However, frequency changes are also expected due to additional forces acting on the sphere, e.g. lift forces and secondary flow, that shift the MM into another shear zone of the quadratic flow; (a) away from the artery wall towards the center of the cross section or (b) vice versa. For case (a) the MM translational motion accelerates and the rotational frequency decreases while in case (b) the MM translational motion decelerates and the rotational frequency increases. Contrary to these shear zone shifts of the MM, reducing the vessel diameter leads to an increase of both, translational motion and rotational frequency. Therefore, knowledge of the actual MM velocity is necessary which is accomplished in MMT by using an array of SQUIDs detecting the spatial magnetic field distribution of a MM. The actual position and orientation of the MM for each measured sample is then determined by solving an inverse problem. In this dipole localization, the most probable MM position and orientation is found that minimizes the residual between measurement data and dipole model. The actual MM velocity is obtained as the first derivative of the MM position with respect to time.

## Results

**Figure 2:**
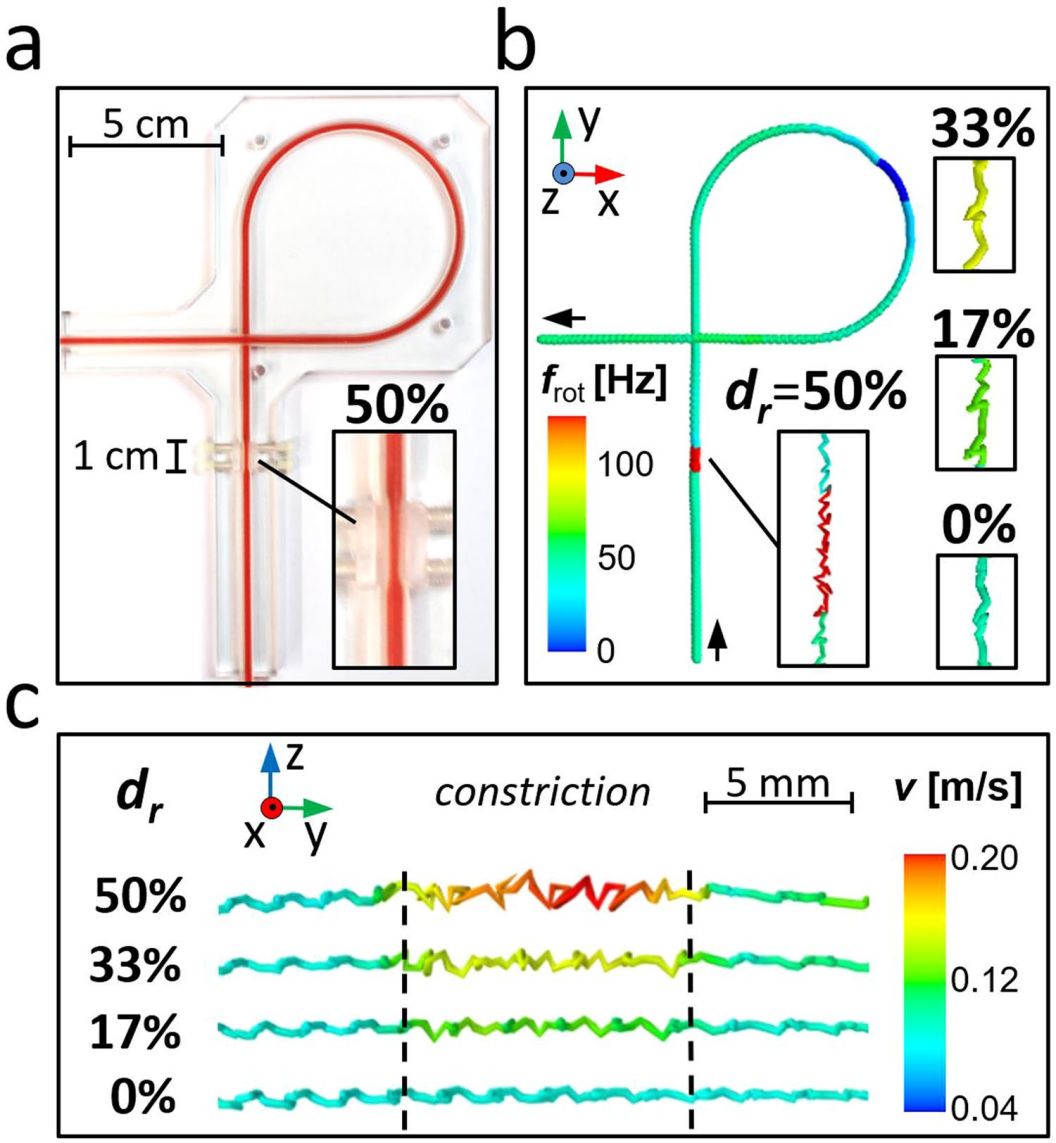
MMT-images for different diameter reductions. (**a**) Photograph of the flow phantom with adjustable constriction region of 1 cm length. It is filled with red ink to visualize the constriction region (shown is 50% diameter reduction *d*_r_). (**b**) Localized *x*, *y*, *z*-positions of MM1 for temporal resolution of 1.3 ms. Color encodes the rotational frequency *f*_rot_ for *d*_r_ 0–50% in a FOV of 15 cm × 20 cm. (**c**) *x*, *y*, *z*-positions (3D lines) of MM1 with the color encoding the actual velocity (*d*_r_ = 0–50%). The flow is directed from left to right and the constriction region is framed by the black dashed lines. We observe that with decreasing constriction diameter the MM’s rotational frequency increases and the sphere accelerates.

Our MMT experiments were performed using a flow phantom as depicted in Fig. 2a. The phantom consisted of a straight tube section, including a variable constriction region (1 cm length) where reductions *d*_r_ up to 50% of the original tube diameter *d*_tube_ of 1.5 mm could be adjusted. Additionally, a curved tube section was chosen to provide a secondary flow. The stray field of an MM passing through the tube was detected by a SQUID array^19^ covering a FOV of 15 cm × 20 cm with a temporal resolution of 1.3 ms. The flow velocity *v*_0_ at the centerline of the tube was adjusted to 0.56 m/s, i.e. close to the upper physiological limit^20^. We used two different MM sizes, MM1 with *d* = 34.6 µm and MM2 with *d* = 5.7 µm. The localized sequence of MM *x*- and y-positions of MM1 is shown in Fig. 2b. The corresponding rotational frequencies are encoded by color. A clear increase of the rotational frequency *f*_rot_ is observed when MM1 passes the constriction while it remains constant in the absence of a constriction. The rotational frequency increases with decreasing flow cross section as displayed in the insets of Fig. 2b (enlargements of the restriction region) for different diameter reductions. As expected, a frequency decrease is observed when MM1 passes the curvature. Here, the secondary flow (Dean effect^21^) shifts the MM’s shear zone towards the tube center.

Figure 2c shows an enlarged view of the MM1 localized positions (*y*-*z* plane) of the constriction passage for different reduction degrees. Here, the color-code represents local velocity *v*. When MM1 enters the constriction it abruptly accelerates, and it decelerates again when it has left the constriction. The MM velocity inside the constriction region increases significantly with decreasing diameter of the flow cross section, while it remains unchanged in the absence of a constriction. This shows the clear impact of the degree of constriction on the localization parameters frequency and velocity. Interestingly, we found small lateral oscillations in the trace of MM1 which slightly increase in amplitude when decreasing the diameter. In the curved section of the phantom (Fig. 2b) the rotational frequencies decrease, and higher velocities than in the constricted regions are observed. To decide whether a constriction or shear zone shift is responsible for the MM acceleration, the velocity-frequency relation is analyzed.

**Figure 3:**
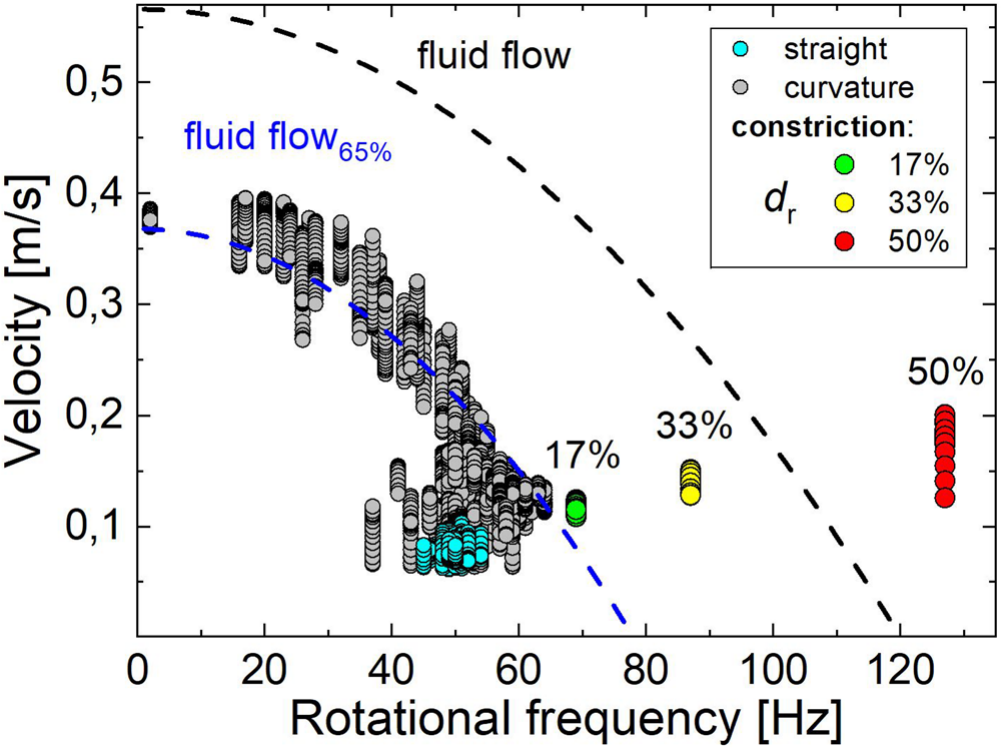
Velocity and rotational frequencies obtained for different regions of the MMT phantom. Velocity of sphere MM1 as a function of the rotational frequency during four individual passages through the FOV of the flow phantom (about 10,000 values). Velocity-frequency combinations are colored cyan in the straight tube section, grey in the curved section, and in the constrained region green for *d*_r_ = 17% reduction, yellow for 33%, and red for 50%, respectively. The dashed black line indicates the theoretical values for velocity *v* and frequency *f*_rot_ of a sphere in quadratic flow *v* = *v*_0_ − [(*πf*_rot_*d*_tube_)^2^/*v*_0_] with *v*_0_ being the fluid velocity in the tube center and the blue line 65% of these values.

In Figure 3, all velocity-frequency combinations within the FOV obtained from four individual passages of MM1 through the phantom are plotted. Combinations obtained in the straight section are colored cyan, those in the curved section grey and those in the constriction regions depending on the reduction degree green (17%), yellow (33%), and red (50%). The black line visualizes the theoretical velocity-frequency relation of the undisturbed parabolic flow and the blue line visualizes the same relation reduced to 65%. In the straight section MM velocity and frequency vary only slightly, and reach values significantly below the undisturbed flow profile. Hence, MM1 lags the undisturbed flow, what is likely due to the higher density of MM1^22^ (seven times higher than the carrier fluid density) and the finite slip velocities near a wall^23^.

Velocities and frequencies show large variations in the curved section of the phantom due to cross-streamline motion of MM1. As expected, there is an inverse correlation between velocity and rotational frequency, i.e. higher velocities are related to lower frequencies. The blue line in Fig. 3 shows the influence of the walls that decelerate MM1. In regions of low frequency, i.e. MM1 being closer to the center of the cross-section, mainly combinations above 65% are obtained, while those combinations obtained when MM1 was closer to a wall are below 65%. In contrast, in the constriction region the highest frequencies are found, and a frequency-velocity relation that is outside this correlation.

An important aspect for a potential biomedical application of MMT is the MM size. Obviously, the diameter should be smaller than the vessel diameter to avoid occlusions. If a MM slightly smaller than red blood cells (6–8 µm) were used, it could pass the pulmonary capillaries in the lungs allowing for MMT monitoring during multiple cycles through the body^16^. On the other hand, the magnetic moment of the MM and thus, its size, must still be high enough to ensure detectability by SQUIDs.

**Figure 4:**
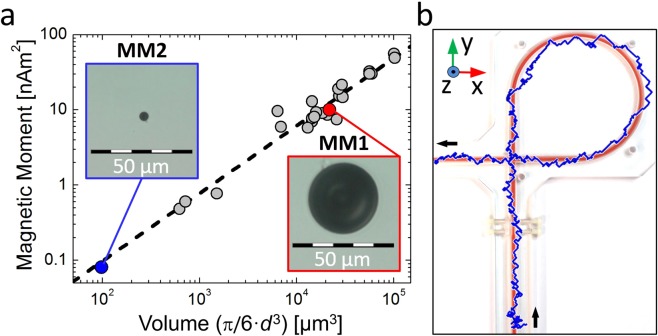
Influence of sphere size in MMT. (**a**) Resulting magnetic dipole moments as a function of MM volume, the red dot denotes sphere MM1 (*d* = 34.6 µm, *m* = 10 nAm^2^), the blue dot denotes sphere MM2 (*d* = 5.7 µm, *m* = 80 pAm^2^). (**b**) Overlay of the flow phantom and localized MMT path obtained for the smallest available sphere, MM2, without constriction.

Figure 4a shows the remnant magnetic moments determined from MMT of spherical MMs as a function of their diameter, after magnetization in magnetic field of 5 T. We find a dipole moment to volume ratio of roughly 0.9 pAm^2^/µm^3^ for the MMs used. Slight deviations from that ratio are ascribed to shape anisotropies (sphericities 0.96–0.99). Figure 4b shows the MMT image obtained for the smallest sphere we used, MM2. With a diameter of only *d* = 5.7 µm it was even smaller than a red blood cell. MM2 exhibits a dipole moment of 80 pAm^2^ generating peak-to-peak amplitudes of about 1 pT (10^−12^ T) at a vertical source to SQUID distance of 3.2 cm. Though the signal-to-noise ratio (SNR) is two orders of magnitude less than for MM1, MMT localizes the path of MM2 through the flow phantom with sufficient accuracy. Hence, MMT imaging is still feasible using a MM smaller than a red blood cell. However, effects due to the reduced SNR become visible by the reduced reconstruction quality, with some MM positions localized outside the tube dimensions.

## Discussion

For clinical application of MMT numerous improvements are possible by hardware, software and protocol adjustments. Several MMs could be injected with a certain distance to each other, and multiple MM passages averaged for SNR improvement. Seven MM2s consecutively injected would allow to image the whole coronary artery tree within a total measurement time of about 15 min. Increasing the number to 70 MM2s (≈18 ng Nd) would then allow to record ten averages. This is based on a 1% probability for the MMs to enter the cardiac vessel of interest and a single pass cycling time of 60 s for each MM through the vasculature. In this scenario, about 10% (i.e. 7 MMs) of the MM2s injected would be simultaneously in the FOV of 20 cm × 20 cm, e.g. the cardiac region, with a mean distance of about 11 cm avoiding clustering. The localization and separation of the seven MM2s is simplified by the following constrains: first, the magnetic moment of each MM is a priori known, reducing the degrees of freedom for each MM to five. Second, the rotational MM frequency depends on the diameter of the actual vessel in the vascular tree. Hence, depending on the progress of each MM to pass the vasculature with decreasing vessel diameters its rotational frequency will steadily increase. Note that this is well distinguishable from a local constriction, where an initial increase of the rotational frequency is followed by an abrupt frequency decrease. Hence, the signal contributions of each MM could be filtered from the measurement data by signal decorrelation techniques (e.g. independent component analysis or wavelets). Considering more than 100 SQUIDs (in theory, 20 localizable MMs) and source decorrelated signals as input for the fitting procedure, the localization and separation of seven MMs becomes realistic. Additional constrains would be possible due to confined MM translation limited by a physiological threshold and its coupling to the rotational MM movement, i.e. a fast MM translation reduces possible MM rotations due to the flow profile and vice-versa.

Further contrast enhancement (more than factor 1.7^24^) is possible by increasing the magnetic moments of the MMs by changing their structural composition. In this respect, also the formation of MM chains that still can pass the smallest capillaries in the lungs would be an option. In our case, a chain of only four MM2s would already lead to signal amplitudes ten times above the measured noise floor of about 400 fT peak-to-peak and thereby increase the localization accuracy. Note that the motion of these MM chains might be more complex following different periodic regimes^25–27^ determined by the local shear flow that need to be considered for blood flow quantification. On the other hand, our present noise level of 3 fT/$\sqrt{{\rm{Hz}}\,}$ may be significantly reduced by hardware optimization, e.g. by low intrinsic noise cryostats. Recent work^28^ reported a noise level of 500 aT/$\sqrt{{\rm{Hz}}\,}$ for frequencies below 1 kHz and even 150 aT/$\sqrt{{\rm{Hz}}\,}$ for frequencies above 10 kHz for their SQUID system. Another promising option for MMT might be the application of optical magnetometers that reduce the MM-to-sensor distance by the omission of cryogenic cooling^29^. By these advancements, SNR improvements of more than two orders of magnitude are expected. This would not only allow an increase in localization accuracy, but also the imaging of deep body regions as e.g. the cardiac region in a depth of approximately 15 cm. MMT imaging of even deeper body regions would be feasible by dedicated sensor arrangements, e.g. positioned circumferentially around the patient. On the other hand, biomagnetic signal contributions of the patient, as e.g. the heart activity known as magnetocardiography, will be visible in the SQUID data. However, these contributions are separable^18^ and could be exploited beneficially by monitoring the patient’s vital functions by MMT allowing to assign the MM movement to the respective cardiac cycle.

In the future, MMT promises outstanding temporal resolution. So far, our proof of concept offers a resolution of 1.3 ms. Since SQUIDs can be operated at bandwidths up to 100 MHz^30^, temporal resolutions in the nanosecond range are accessible by MMT.

Further studies are needed to investigate MM interactions with artery walls and blood cells. For the latter, Das *et al*. observed lateral oscillations of microspheres in pipe flow by microscopy that he referred to an increase of the hematocrit level, i.e. red blood cells^31^. They observed that these lateral oscillations contain higher harmonics of the rotational frequency^31^. Though the lateral oscillatory MM traces (cf. Fig. 2c) were unwanted in our experiment, we demonstrated that localization and classification of constricted volumes is feasible in the presence of such traces. In our case, the lateral oscillations are also visible as multiples of the rotational MM frequency directly in the SQUID data and might be a consequence of flow disturbances created by the MM^32^ or an imbalance between hydrodynamic and non-hydrodynamic (e.g. gravity) forces.

We demonstrated the tracking of a single microsphere (the size of a red blood cell) with a temporal resolution of 1.3 ms and a spatial resolution below 1 mm within a FOV of 15 cm × 20 cm. From the measurements we were able to quantify the flow conditions experienced by the microsphere. A unique feature of MMT is the independent monitoring of rotational and translational MM velocities. This enables localization and classification of constricted volumes and distinction from cross-streamline motion of the MM. Additionally, separating influences of Stokes friction and shear flow on a sphere makes MMT an ideal tool for rheology investigations. The fast and sensitive MMT flow monitoring allows the observation of lateral oscillatory MM trajectories across streamlines and might help to understand complex flow dynamics.

MMT has the potential to become a straightforward method for blood flow quantification offering some advantages over the aforementioned modalities. Obviously, a single sphere represents an ideal “sharp” bolus for blood flow quantification that is not hampered by dispersion effects. The quantitative parameters on hemodynamics obtained by MMT are independent from the presence of bones, air or tissue and are therefore reliably interpreted to sensitively indicate flow perturbations in arteries. The excellent temporal resolution of MMT is as fast as obtained in coronary angiography. At the same time, MMT provides true 3D information at high resolution, which is currently only accessible using slower tomographic methods like CT or MRI. As MMT is a noninvasive method that operates without radiation or strong magnetic fields, it may be suitable for screening applications. The applied quantity of tracer material (in our experiment 0.25 ng of neodymium (Nd)) is multiple orders of magnitude below the dose limit of 70 µg/kg^33^. Hence, clinical MMT could improve the diagnosis of cardiovascular diseases and pave the way for a better understanding of the hemodynamics of the arterial system.

## Methods

### Hardware and magnetic microspheres

Magnetic Tracking of the MMs was performed inside a magnetically shielded room (BMSR2^34^) using the PTB 304 SQUID vector magnetometer system^19^. In this system, a number of 304 SQUIDs are distributed over four horizontal layers detecting the three components of the magnetic flux density **B** = [*B*_x_, *B*_y_, *B*_z_]. Data was recorded with a sampling frequency *f*_s_ = 750 Hz. The flow phantom (cf. Fig. 2) was placed in a vertical distance of 3.5 cm (MM1) and 3.2 cm (MM2) to the closest SQUID. It comprised a syringe pump (Landgraf® LA800), a silicone tube of inner diameter *d*_*tube*_ = 1.5 mm (150 ml total volume) and an outlet container. Purified lamp oil (BOOMEX®, viscosity *η* = 2 mm^2^/s) served as flow medium and was pumped with a flow rate of 30 ml/min, i.e. an average fluid velocity of 0.28 m/s, resulting in an intermediate flow regime of Reynolds number *Re* = 212. The magnetic microspheres MM1 and MM2 were separated from commercial Magnequench® powder (MQP™-S-11-9) using a cell selector (ALS®). The selector allowed to monitor the separation process and estimate the size of the MMs via microscopy. After separation, each MM was magnetized at a flux density of 5 T inside a magnetic property measurement system (MPMS – Quantum Design®).

### Reconstruction principle and used constrains

The magnetic flux density *B*_sim_ generated by the MM’s magnetic moment **m** = [*m*_x_, *m*_y_, *m*_z_] at a location **r**′ = [*r*_x_′, *r*_y_′, *r*_z_′] detected by a SQUID at position **r** = [*r*_x_, *r*_y_, *r*_z_] with a sensor normal **n** = [*n*_x_, *n*_y_, *n*_z_] is given by the well-known formula of a magnetic dipole.

1 $${B}_{sim}=\frac{{\mu }_{0}}{4{\rm{\pi }}}(\frac{3{{\bf{n}}}^{{\rm{T}}}(({\bf{r}}-{\bf{r}}{\boldsymbol{^{\prime} }})\cdot {({\bf{r}}-{\bf{r}}{\boldsymbol{^{\prime} }})}^{{\rm{T}}})}{|{\bf{r}}-{\bf{r}}{\boldsymbol{^{\prime} }}{|}^{5}}-\frac{{{\bf{n}}}^{{\rm{T}}}}{|{\bf{r}}-{\bf{r}}{\boldsymbol{^{\prime} }}{|}^{3}}){\bf{m}},$$

with *µ*_0_ being the magnetic constant. In spherical coordinates, the vector components of **m** are described by

2 $${m}_{x}=m\cdot \,\sin (\theta )\cdot \,\cos (\varphi )$$

$${m}_{y}=m\cdot \,\sin (\theta )\cdot \,\sin (\varphi )$$

$${m}_{z}=m\cdot \,\cos (\theta )$$

with total magnetic moment *m* = |**m**|, horizontal angle *θ* and vertical angle *ϕ* with respect to the *x*, *y*, and *z*- axis. The reconstruction of the location of **m** and its orientation defined by the two angles (*θ*, *ϕ*) based on the vector containing the measurement data of *P* (=*133*) SQUIDs **B**_meas_ = [*B*_1_, ..., *B*_p_] is an inverse problem. This inverse problem can be solved by fitting the field of a magnetic dipole (Eq. (1)) to the measured magnetic field components. Hence, minimizing the cost function.

3 $${\rm{\Omega }}({\bf{r}}{}^{{\boldsymbol{^{\prime} }}},\theta ,\varphi )={\rm{a}}{\rm{r}}{\rm{g}}{\rm{m}}{\rm{i}}{\rm{n}}{\Vert {{\bf{B}}}_{{\rm{m}}{\rm{e}}{\rm{a}}{\rm{s}}}-{{\bf{B}}}_{{\rm{s}}{\rm{i}}{\rm{m}}}\Vert }^{2}$$

To reconstruct the MM path through the vessel, this dipole localization is performed for each sample *i* of the measurement **B**_meas_(*I*). Thus, to reconstruct 750 MM locations and orientations obtained within a second, an efficient algorithm for minimizing this cost function is mandatory. We found the Levenberg-Marquardt algorithm^35,36^ as a combination of a steepest slope iteration with an iteration according to the inverse Hessian matrix to perform best. It was previously applied for magnetic marker monitoring^18^. The total magnetic moment *m* (*m*_MM1_ = 10 nAm^2^, *m*_MM2_ = 80 pAm^2^) was kept constant and constrains were set to limit the MM translational movement in the model from one sample to another

4 $${{\bf{r}}{}^{{\boldsymbol{^{\prime} }}}}_{i+1}={{\bf{r}}{}^{{\boldsymbol{^{\prime} }}}}_{i}\pm \sum $$

within the confined search region Σ allowing for a maximum MM velocity of *v*_max_ = 2 m/s in each direction.

### Estimation of MM velocity and rotational frequency

The actual MM velocity *v*_*i*_ was estimated for sample *I* − 1 as first derivative of **r**’ by

5 $${v}_{i}=|{{\bf{r}}{\boldsymbol{^{\prime} }}}_{i}-{{\bf{r}}{\boldsymbol{^{\prime} }}}_{i-1}|\cdot {f}_{s}$$

multiplied by the sampling frequency *f*_s_ = 750 Hz. Additionally, *v*_*i*_ was smoothed by a moving average with a width of 10 samples to reduce velocity variations due to lateral MM movements (oscillations). The rotational frequency of the MM *f*_rot_ over time was estimated in two steps: first, a frequency *f*_pre_ was estimated by analysis of the spectrogram of the *θ*, *ϕ* traces. Second, *f*_pre_ served to adaptively adjust a window of width *W* = *2*(*f*_s_*/f*_pre_) to consecutively estimate *f*_rot_ by fitting a sine wave to the *θ*, *ϕ* traces.

## Supplementary information

Real-time Magnetic Microsphere Tracking

## Data Availability

Data supporting the findings of this manuscript are available from the corresponding author upon request.
